# *Schizochytrium* sp. Extracted Lipids Prevent Alopecia by Enhancing Antioxidation and Inhibiting Ferroptosis of Dermal Papilla Cells

**DOI:** 10.3390/antiox12071332

**Published:** 2023-06-23

**Authors:** Zuye Zeng, Boyu Wang, Muhammad Ibrar, Ming Ying, Shuangfei Li, Xuewei Yang

**Affiliations:** 1 Guangdong Technology Research Center for Marine Algal Bioengineering, Guangdong Key Laboratory of Plant Epigenetics, College of Life Sciences and Oceanography, Shenzhen University, Shenzhen 518060, China; 2100251019@email.szu.edu.cn (Z.Z.); yingming@szu.edu.cn (M.Y.); sfli@szu.edu.cn (S.L.); 2 School of Life Science and Technology, Wuhan Polytechnic University, Wuhan 430023, China; 3 Innova Bay (Shenzhen) Technology Co., Ltd., Shenzhen 518118, China

**Keywords:** alopecia, unsaturated fatty acids, *Schizochytrium* sp. extracted lipids, dermal papilla cells, antioxidant, ferroptosis

## Abstract

Alopecia has gradually become a problem that puzzles an increasing number of people. Dermal papilla cells (DPCs) play an important role in hair follicle (HF) growth; thus, exploring the effective chemicals or natural extracts that can remediate the growth of DPCs is vital. Our results showed that *Schizochytrium* sp.-extracted lipids (SEL) significantly promoted proliferation (up to 1.13 times) and survival ratio (up to 2.45 times) under oxidative stress. The treatment with SEL can protect DPCs against oxidative stress damage, reducing the reactive oxygen species (ROS) level by 90.7%. The relative gene transcription and translation were thoroughly analyzed using RNA-Seq, RT-qPCR, and Western blot to explore the mechanism. Results showed that SEL significantly inhibited the ferroptosis pathway and promoted the expression of antioxidant genes (up to 1.55–3.52 times). The in vivo application of SEL improved hair growth, with the length of new hair increasing by 16.7% and the length of new HF increasing by 92.6%, and the period of telogen shortening increased by 40.0%. This study proposes a novel therapeutic option for alopecia, with the effect and regulation mechanism of SEL on DPC systematically clarified.

## 1. Introduction

Alopecia is a dermatologic disorder in which sudden or gradual hair loss occurs in one or more body areas [1]. With the increase in multiple pressure factors, such as the intensification of pollution, the number of people suffering from alopecia is gradually rising [2]. Dermal papilla cells (DPCs) preserved under hair follicles play an essential role in postnatal hair growth and hair follicular morphogenesis [3]. DPCs are a reservoir of growth factors, nutrients, cytokines, and multi-potent stem cells, thereby regulating hair follicle growth and development [4]. In postnatal life, the growth cycle of hair follicles undergoes catagen and cyclical anagen stages [5]. During the catagen growth stage, the epithelial cells underlying the follicle cells undergo apoptosis, while that of DPC remains intact and grows upwards alongside hair follicle stem cells. At the anagen stage, DPCs activate the stem cells of secondary hair germs, leading to a downward-facing growth of new follicles. Exploring the effective chemicals or natural extracts that can remediate and stimulate the upward growth of DPCs, which is crucial for alopecia treatment.

Oxidative stress via the overproduction of free radicles or an inefficient antioxidant defense system is one of the leading factors causing alopecia [6,7,8]. Human skin is continuously exposed to various environmental and endogenous pro-oxidant agents that produce reactive oxygen species (ROS), thereby leading to cellular constituent damage, including cell membrane lipids, proteins, and nucleic acids [9,10]. Currently, only two drugs (finasteride and minoxidil) are available in the market, and they are approved by the US Food and Drug Administration (FDA) [11]. Although these drugs promote hair growth and reduce hair loss, serious side effects are the main drawbacks for broader applications [12,13]. For example, minoxidil can cause contact dermatitis and hirsutism [14], and finasteride can increase the incidence of sexual dysfunction [15]. Given the limited efficacy, finding new natural anti-hair loss ingredients that are less harmful and more effective is significant. Recently, natural extracts, such as herbal medication, have been proposed for promoting hair growth and preventing hair loss. In particular, applying these extracts has many advantages, including low cost, fewer side effects, patient compliance, easy availability, and more than one mode of biochemical action [16]. The research on natural drugs that promote the growth of DPCs mainly focus on some plant extracts, such as *Carthamus tinctorius* floret extracts [17], *Crataegus pinnatifida* extracts [18], *Geranium sibiricum* extracts [19], and *Polygonum multiflorum* extracts [20]. In addition, some natural compositions were obtained from animals, such as human placental extracts [21] and phospholipids purified from porcine lung tissues [22]. However, there are few studies on the effect of the microalgal extract on DPCs. Green algae strain *Mucidosphaerium* sp. isolated from a hot spring can downregulate the gene expression level of proinflammatory cytokines in DPCs [23]. The methanol extract of edible seaweed *Ecklonia cava* can elongate the hair stem in cultured human hair follicles and promote the transition of the hair cycle of the back skin of C57BL/6 mice from the resting to the growth stage. In addition, it also induces IGF-1 expression in DPCs cells [24]. 7-phloroeckol isolated from *Ecklonia cava*, one of the marine brown algae, has been proven to promote DPC proliferation [25]. Polyunsaturated fatty acids (PUFAs) are an essential precursor for various functions in the human body [26]. Microalgae are one of the important sources of PUFAs. Although fish oil contains a large number of PUFAs, fish cannot synthesize PUFAs by themselves. Fish accumulate PUFAs mainly by eating microalgae. There are two major classes of PUFAs: ω-3 PUFAs and ω-6 PUFAs. ω-3 PUFAs include α-linolenic acid (18:3; ω-3; α-LA), eicosapentaenoic acid (20:5; ω-3; EPA), and docosahexaenoic acid (22:6; ω-3; DHA) [27]. Compounds that are structurally similar to ω-3 PUFAs, such as latanoprost, isopropyl unoprostone, and bimatoprost, induce hair growth in both mice and humans [28,29]. Moreover, a mackerel-derived fermented fish oil (FFO) extract and its component DHA improve hair growth via DPC anagen-activating pathways by inducing cell cycle progression and activating extracellular signal-regulated kinase (ERK), p38, and Akt [30]. Arachidonic acid (AA), a structurally similar compound to ω-6 polyunsaturated fatty acid, was found to promote DPC’s viability and enhance fibroblast growth factor-7 (FGF-7) and FGF-10 [31] expression, thereby promoting hair growth. In addition, an ex vivo hair follicle culture shows that AA considerably promotes hair shaft elongation, increasing matrix keratinocyte proliferation [32]. Moreover, the linoleic acid extracted from *Malva verticillata* seeds activates Wnt/β-catenin signaling and induces DPC growth [33]. The efficient natural source alternative to fish for ω-3 long-chain polyunsaturated fatty acids (LC-PUFAs) is the unicellular fungus-like protist *Schizochytrium* sp. [34]. Since microalgae have a slightly fishy smell, fast growth rate, high purity, and high DHA content, utilizing *Schizochytrium* sp. for PUFA production could pave its way to the market [35]. However, there is a lack of reports on the effect and regulation mechanism of unsaturated fatty acids from *Schizochytrium* sp. on DPCs.

To investigate the effect and regulation mechanism of *Schizochytrium* sp. extracted lipids (SEL) on DPCs, cell proliferation efficiency and antioxidant capacity were analyzed in the current study. Moreover, the regulation of the critical signaling pathways related to growth and antioxidation in DPCs affected by SEL was also explored by comparing the transcriptome and protein expression. To verify the effect of SEL on promoting hair growth, the time of follicle telogen, length of new hair and follicle, and weight of new hair of C57BL/6 mice were measured. This investigation helps clarify the effect and regulation mechanism of SEL on DPCs and provides the possibility to promote hair growth and reduce hair loss by enhancing the growth of DPCs and improving their antioxidant capacity.

## 2. Materials and Methods

### 2.1. Reagents

SEL was purchased from Innova Bay (Shenzhen) Technology Ltd. (Shenzhen, China). SEL was extracted as follows: freeze-dried cells of *Schizochytrium* sp. INNOVA23 were placed in a pretreated filter paper bag as a filter paper package and extracted with methanol in a Soxhlet extractor at 60 °C for 72 h. The extracted liquid was evaporated to dryness at 60 °C by using a rotary evaporator.

Dimethyl sulfoxide (DMSO), ethanol, methanol, sulfuric acid, hydrogen peroxide (H_2_O_2_), dichloromethane, formalin buffer, xylene, and hematoxylin, and eosin were purchased from Macklin (Shanghai, China). Dulbecco’s Eagle medium (DMEM) and fetal bovine serum (FBS) were purchased from Hyclone (Logan, UT, USA). CCK-8, minoxidil, and 2′,7′-Dichlorodihydrofluorescein Diacetate (DCFH-DA) were purchased from MCE (Shanghai, China). The TRIzol reagent, RIPA lysis buffer, protease inhibitor, and BCA analysis kit were purchased from Solarbio (Beijing, China). PrimeScript™ RT reagent Kit and TB Green^®^ Premix Ex Taq™ II were purchased from Takara (Dalian, China). The PVDF membrane was purchased from Beyotime (Shanghai, China). A chemiluminescent substrate kit was purchased from Mei5bio (Beijing, China).

### 2.2. Composition Analysis of SEL

The composition of SEL was analyzed according to a previously reported method [36]. In short, 100 mg of SEL was mixed with 2 mL of 4% methanol and sulfuric acid and heated in a water bath at 65 °C for 1 h in order to obtain fatty acid methyl esters (FAMEs). Afterward, 1 mL of both n-hexane and deionized water was added to FAMEs, and the upper organic phase was taken after shaking. Subsequently, the organic solvent in the liquid was thoroughly blown dry to obtain methyl-esterified fatty acids (MEFs). MEFs were finally dissolved in dichloromethane (500 μL) for gas chromatography–mass spectrometry (GC-MS) (Agilent, Santa Clara, CA, USA) analysis. An HP-5MS GC column (30.0 m × 250 µm, Agilent, Santa Clara, CA, USA) was used to analyze FAMEs. In this study, 1 µL of each FAMEs sample was injected into the column. The constant pressure mode was used, and the shunt ratio was 10:1, with helium as a carrier gas. The column temperature increased from 60 °C to 180 °C at a constant rate of 25 °C per min and then increased to 240 °C at a rate of 3 °C per min, remaining at 240 °C for 1 min and finally increasing to 250 °C at a constant rate of 5 °C per min. Mass spectrometry was performed using full-scan mode detection. Fatty acids were identified using the mass spectrometry library of the National Institute of Standards and Technology (NIST).

### 2.3. Cell Viability Assay

DPCs were purchased from MeisenCTCC (Hangzhou, China). DPCs were cultured in DMEM containing 10% FBS and kept under 37 °C at 5% CO_2_. SEL was dissolved in DMSO/ethanol (1:1, *v/v*) at a concentration of 50 mg mL^−1^, and the DMSO/ethanol solution used was 0.2% or less than the culture medium [30]. The SEL’s effect on DPC cell viability was monitored using a CCK-8 assay according to the manufacturer’s protocol [37]. Briefly, DPCs (3 × 10^3^ cells/well) were cultured for 24 h in 96-well plates. After incubation, the cells were treated with minoxidil (12.5 µg mL^−1^), different concentrations of SEL (100, 50, 25, 12.5, 0 µg mL^−1^), and solvent, and then they were cultured for 12, 24, and 36 h, respectively. The concentration selection of SEL is based on a previously reported study [30]. After the culture, before adding the CCK-8, each well was washed with D-PBS. Afterward, the 96-well plates were kept in a humidified incubator at 37 °C for 2 h with 5% CO_2_. Finally, the absorbance (450 nm) was measured to record the effect. Each sample was repeated three times for statistical analysis.

### 2.4. Antioxidant Capacity Assay

The SEL effect on DPCs’ antioxidant capacity was examined by using a CCK-8 assay. The cells were treated with 100 μM of H_2_O_2_ for 1.5 h [38]. The cells’ survival and toxicity in a H_2_O_2_-containing medium were monitored by a CCK-8 assay, as described above. The antioxidant capacity was expressed by comparing the cell viability of DPC treated with the same drug before and after oxidative stress.

### 2.5. 2′,7′-Dichlorodihydrofluorescein Diacetate Assay for Detecting the ROS Level

To observe the effect of SEL on DPCs at the level of ROS, the ROS fluorescent probe DCFH-DA was utilized to detect the cells’ ROS levels [39]. 2′,7′-dichlorodihydro-fluorescein (DCFH) dye was oxidized to form a fluorescent 2′,7′-dichlorofluorescein (DCF) substance in the presence of ROS, and the intensity of green fluorescence was directly proportional to the ROS level inside cells [40]. After drug treatment and oxidative stress, DCFH-DA (5 µM) was added to each well that contained cells, and the cells were incubated in the dark at 37 °C for 30 min. After incubation, the wells were washed twice with fresh D-PBS to remove the residual probe. Then, pictures of cells were taken using an inverted fluorescent microscope (Leica, Weitzlar, Germany). ImageJ V.1.8.0 (National Institutes of Health, Bethesda, MD, USA) was used to calculate fluorescence intensity. In short, the fluorescence image was first converted into an 8-bit black and white image, and then the average grayscale value was measured at a certain threshold to represent the average fluorescence intensity [41].

### 2.6. RNA-Sequencing Analysis

Groups with the most significant effect were sent to Beijing Baimaike Biotechnology Co., LTD for transcriptome sequencing. DESeq and *p*-value were employed and used to evaluate differential gene expression. Here, only genes with |log2 FC| ≥ 1, where FC denotes the fold change and *p* < 0.05, were used for subsequent analyses [42]. To annotate the main biological functions of differentially expressed genes (DEGs), gene ontology (GO) analysis was conducted by using the BMKCloud V.2022 (Biomarker Technologies, Beijing, China). Kyoto Encyclopedia of Genes and Genomes (KEGG) analysis was used to identify signaling pathways based on DEG using BMKCloud V.2022 (Biomarker Technologies, Beijing, China).

### 2.7. Real-Time Quantitative PCR (RT-qPCR) Analysis

RT-qPCR was utilized to explore key gene expression levels. Total RNA was extracted from the cells treated with different drugs using the TRIzol reagent. NanoDrop 2000 (Thermo Scientific, Waltham, MA, USA) was used to measure the purity and concentration of RNA. When A260/280 > 2.0 and A260/230 > 2.0, this indicates that RNA purity is high and can be used for further experiments. The PrimeScript™ RT reagent Kit was used to synthesize cDNA. TB Green^®^ Premix Ex Taq™ II and ABI QuantStudio 6 Flex (Applied Biosystems, Foster, CA, USA) were used for RT-qPCR according to the manufacturer’s protocol. Primer sequences used for RT-qPCR are listed in Supplementary Materials (Table S2). The cycling protocol was as follows: 1 cycle at 95 °C for 30 s, followed by 40 cycles at 95 °C for 5 s and at 60 °C for 30 s, and then followed by 1 cycle at 95 °C for 15 s, at 60 °C for 1 min, and at 95 °C for 15 s. The relative gene expression was calculated as 2^^−△△Ct^ using ACTB as the housekeeping gene for normalization.

### 2.8. Western Blot Analysis

DPCs with a density of 6.0 × 10^5^ were seeded into 6-well plates. After pre-incubation for 24 h, the DPCs were treated with SEL (50 µg mL^−1^) for 36 h. Western blot analysis was performed as described before [43]. Briefly, DPC was washed with PBS and then cleaved on ice for 30 min with a 100 μL RIPA lysis buffer containing 1% protease inhibitor. After centrifugation at 13,000 rpm for 15 min, the supernatant was collected for measuring the protein concentration using a BCA analysis kit. The protein of equal weight (10 μg) was electrophoresed in 12% SDS-PAGE gel and transferred to a PVDF membrane. After being enclosed with 5% skim milk for 1 h at room temperature, the membrane was incubated with the primary antibody overnight (Table S3) at 4 °C and 1 h with the secondary antibody at room temperature. Protein bands were displayed using the chemiluminescent substrate kit and the ChempChemiTM+ system (SageCreation, Beijing, China). The density of each band was quantified by ImageJ V.1.8.0 (National Institutes of Health, Bethesda, MD, USA).

### 2.9. Hair Growth Activity In Vivo

Seven-week-old healthy C57BL/6 mice, weighing 18–22 g, were obtained from the Center for Disease Control of Hubei province (Wuhan, China). The mice were kept under the following conditions: temperature at 23 ± 2 °C, photoperiod comprising 12 h light:12 h darkness, and humidity at 35–60%. The diet was consumed ad libitum except for the gavage group. After a one-week laboratory acclimation period, mice were anesthetized using 1% pentobarbital sodium by intraperitoneal injection, and then all mice’s backs were shaved with a razor blade. The experiment was divided into 6 groups, with 5 mice in each group, namely, the control, negative control, 5% minoxidil group, SEL-smeared group (5% and 10%), and SEL-gavage group (5%). SEL was dissolved in a 1:1 ratio of propylene glycol/ethanol, and without SEL, ethanol/propylene glycol was used as a negative control. Each mice group was treated for a total of 15 days (once a day). The changes in hair growth were recorded on days 0, 5, 10, and 15. On the 15th day, the new hairs on the mice’s backs were scraped to measure the weight and hair length. All animal experiments were carried out according to the NIH Guideline for the Care and Use of Laboratory Animals and approved by the Animal Ethical and Welfare Committee of Shenzhen University (AEWC-202200008).

### 2.10. Morphological Analysis of Hair Follicles by HE Stains

CO_2_ overdose by inhalation was used to euthanize mice on the 15th day. When the mice did not move, did not breathe, and had dilating pupils, CO_2_ was turned off. After turning off CO_2_, we carried out observations for 2 min to confirm the mouse’s death. Afterward, 1 cm^2^ skin pieces from the center of the treated area were removed and fixed in a 10% formalin buffer. Flowing water was used to remove the fixative for 20 min. Then, the skin slices were dehydrated using an ethanol gradient (50% to 100%) and soaked in xylene for cleaning. The skin sections were then soaked in paraffin to achieve a uniform thickness of 10 μm. Finally, the pieces of skin were stained with hematoxylin and eosin (H&E) [17]. Changes in hair follicles were observed using 100× magnification for the field of vision. The three longest hair follicles in the field of vision were selected for calculations in order to compare hair follicle lengths.

### 2.11. Statistical Analysis

All data are expressed as the mean value ± standard error of the mean (SEM) and analyzed by the Prism 9.0 (GraphPad Software, San Diego, CA, USA). One-way ANOVA and unpaired *t*-tests were used to evaluate the differences between different treatments. A *p* value of < 0.05 was considered statistically significant.

## 3. Results

### 3.1. Composition Analysis of SEL

**Figure 1:**
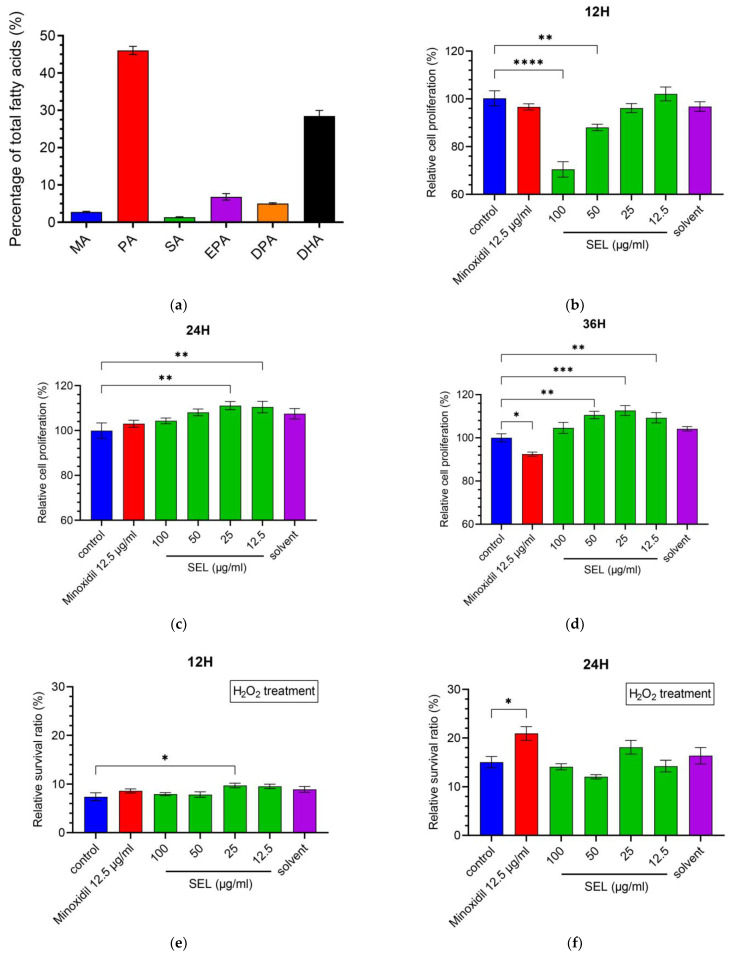

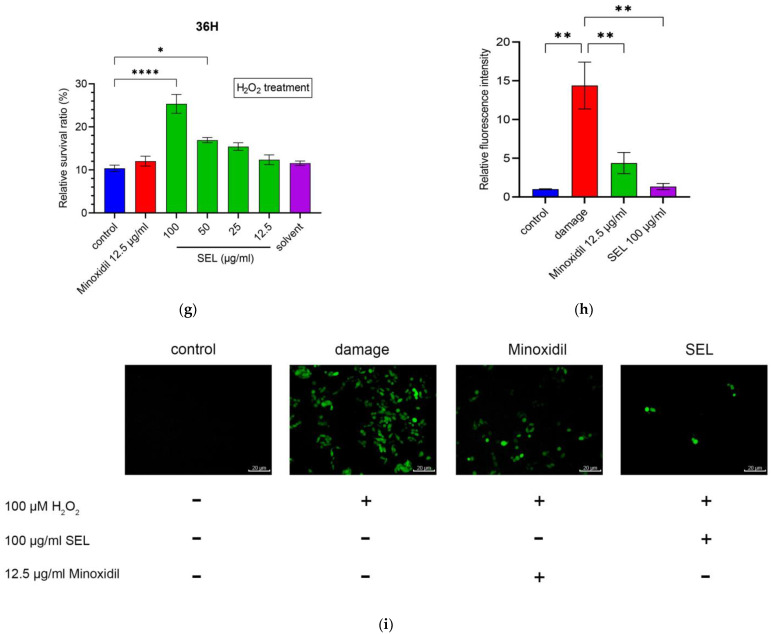
SEL promoted the proliferation and antioxidant capacity of DPC. (**a**) Composition of SEL. MA, Myristic acid (C_14_H_28_O_2_); PA, palmitic acid (C_16_H_32_O_2_); SA, stearic acid (C_18_H_36_O_2_); EPA, eicosapentaenoic acid (C_20_H_30_O_2_); DPA, docosapentaenoic acid (C_22_H_34_O_2_); DHA, docosahexenoic acid (C_22_H_32_O_2_). (**b**–**d**) DPC cell proliferation exposed to SEL (12.5, 25, 50, and 100 µg mL^−1^) is shown at 12, 24, and 36 h. Minoxidil as a positive control and solvent (0.2% DMSO/ethanol (1:1)) as a negative control were used. (**e**–**g**) The survival ratio of DPC exposed to H_2_O_2_ (100 µM) for 1.5 h. (**h**) The summary graph of the relative intensity of fluorescence on oxidatively damaged DPCs. (**i**) The images of the DCFH-DA probe fluorescence of oxidatively damaged DPCs. Control, no treatment; damage, treated with 100 µM H_2_O_2_; minoxidil, treated by 12.5 µg mL^−1^ minoxidil + 100 µM H_2_O_2_; SEL, treated with 100 µg mL^−1^ SEL + 100 µM H_2_O_2_. Data are presented as the mean ± standard error of the mean (SEM) (*n* = 3). * *p* < 0.05; ** *p* < 0.01; *** *p* < 0.001; **** *p* < 0.0001 vs. control.

The main components of SEL are palmitic acid (PA) (C_16_H_32_O_2_) (46.1%), DHA (C_22_H_32_O_2_) (28.5%), EPA (C_20_H_30_O_2_) (6.8%), docosapentaenoic acid (DPA) (C_22_H_34_O_2_) (5.0%), myristic acid (MA) (C_14_H_28_O_2_) (2.8%), and stearic acid (SA) (C_18_H_36_O_2_) (1.4%). The SELs are composed of unsaturated and saturated fatty acids at a percentage of 40.3% and 50.2%, respectively (Figure 1a). The content of DHA in SEL was as high as 28.5%, indicating that *Schizochytrium* sp. is one of the high-quality sources of DHA.

### 3.2. SEL Promoted the Proliferation of DPC

DPCs play essential roles in hair cycle regulation. To investigate whether SEL could promote hair growth, the CCK-8 assay was utilized to measure the SEL effect on the proliferation of immortalized DPCs (Figure 1b–d). Compared with control, SEL significantly promoted the proliferation of DPC at 24 h (an increase of 10.5%–11.1%) and 36 h (an increase of 9.3%–12.6%). In particular, the proliferation ratio of DPC treated with various concentrations (12.5, 25, 50, and 100 µg mL^−1^) of SEL was 1.13–1.22 times that of the DPC treated with minoxidil at 36 h (Figure 1d). These results suggested that SEL was beneficial for DPC growth after co-cultivation for 24 h, indicating its positive effect on stimulating hair growth by inducing DPC proliferation.

### 3.3. SEL Enhanced the Antioxidant Capacity of DPC

Oxidative stress and subsequent DNA damage are crucial for monitoring alopecia [44]. Therefore, DPCs were exposed to H_2_O_2_ after co-cultivation with SEL to explore the influence of SEL on antioxidant capacity. Results showed that 100 µM H_2_O_2_ resulted in 84.9% to 92.6% death for DPCs after exposure for 1.5h (Figure 1e–g). It is worth noting that, at 36 h, SEL (100 µg mL^−1^) significantly improved the antioxidant capacity of DPC, with the cell survival ratio reaching up to 25.4%, which was 2.45 times and 2.11 times higher compared with the control and minoxidil-treated group (Figure 1g).

Excessive ROS or free radicals may induce hair follicle aging and developmental disorders of the hair [45,46,47]. The level of ROS in DPCs was detected using a DCFH-DA active oxygen fluorescence probe. Figure 1i represents the intensity of the cells’ green fluorescence active oxygen level. After the oxidative stress treatment, the fluorescence intensity of the damaged group was 14.39 times that of the control group, indicating that H_2_O_2_ increased the level of ROS in DPCs. Notably, the fluorescence intensity of the minoxidil group and SEL group was only 30.4% and 9.3% of the damage group (Figure 1h), respectively. In conclusion, results suggested that compared with minoxidil, SEL showed an impressive advantage: it greatly reduced the level of ROS caused by oxidative stress.

### 3.4. Comparative Transcriptome Analysis for Various Treatment

Since 50 µg mL^−1^ of SEL showed significant effects in promoting proliferation and antioxidation at 36 h, DPCs with no treatment (C), H_2_O_2_ treatment (CH), 50 µg mL^−1^ SEL treatment (CS), and 50 µg mL^−1^ SEL + H_2_O_2_ treatment (CHS), after culturing for 36 h, were sent for transcriptome sequencing to study the mechanism of SEL.

#### 3.4.1. Quality Analysis of Transcriptome Data and Statistics of DEGs

**Figure 2:**
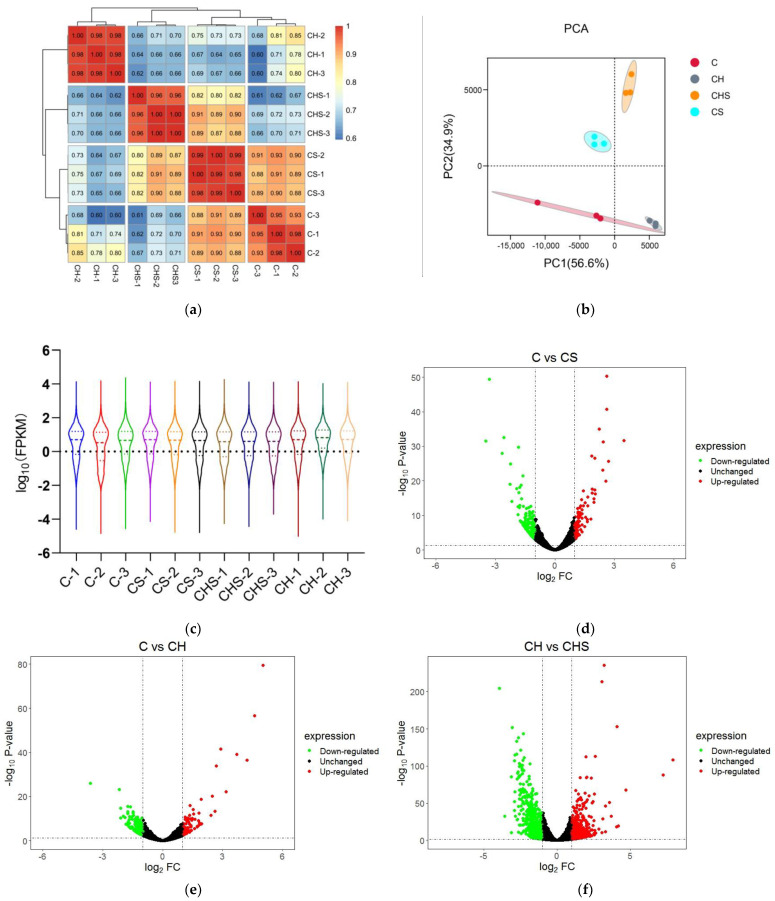

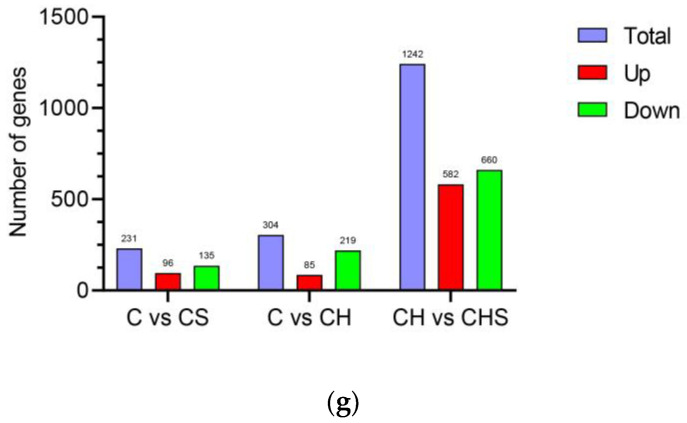
Quality assessment of RNA-seq data. (**a**) Pearson’s correlation analysis based on logarithm-transformed counts from the RNA-seq dataset of C, CS, CH, and CHS. (**b**) PCA analysis. The PCA axis percentage represents the proportional variance of each PC interpretation, and the upper–right side of the diagram represents the conditions. (**c**) Violin plot indicating gene expression and visualizing genes data density in each sample. (**d**–**f**) The volcano plot of DEGs in C vs. CS (**d**), C vs. CH (**e**), and CH vs. CHS (**f**). The gene expression fold change values on log2 (x-axis) and the significance of differential expression *p*-value on -log10 (y-axis). The vertical bar is twice the difference threshold, and the horizontal line is the *p* = 0.05 threshold. Red dots (upregulated), blue dots (downregulated DEGs), and black dots (non-DEG). (**g**) The summary graph of DEG in three groups. C, cell without treatment; CS, cell treated with 50 µg mL^−1^ SEL; CH, cell treated with 100 µM H_2_O_2_; CHS, cell treated with 50 µg mL^−1^ SEL + 100 µM H_2_O_2_.

The sequencing results showed that the percentage of Q30 bases was 94.0% or more, and the comparison efficiency between each sample’s clean reads and the specified reference genome was 94.7% to 96.8% (Table S1). Furthermore, Pearson’s correlation analysis showed that samples within the same group were highly similar at *R*^2^ = 0.93~1.00, while samples in different groups were distinct (Figure 2a). The principal component analysis (PCA) score chart showed that four groups formed, showing precise classification among C, CS, CH, and CHS (Figure 2b). The violin plot showed that interquartile spacing, median, and gene expression distribution were nearly identical within the groups, indicating the high reproducibility of parallel samples (Figure 2c). These results demonstrate the transcription features’ separation among four groups, suggesting that RNA-seq data could be utilized for subsequent downstream analysis.

RNA-Seq data were used to find DEGs, and the gene expression levels with *p*-values of <0.05 and |log2 FC| ≥ 1 were considered as exhibiting a significant difference. A total of 1777 DEG genes were identified, with 96, 85, and 582 upregulated DEGs and 135, 219, and 660 downregulated DEGs in C vs. CS, C vs. CH, and CH vs. CDH groups, respectively (Figure 2d–g).

#### 3.4.2. Functional Analysis by GO and KEGG Enrichment

**Figure 3:**
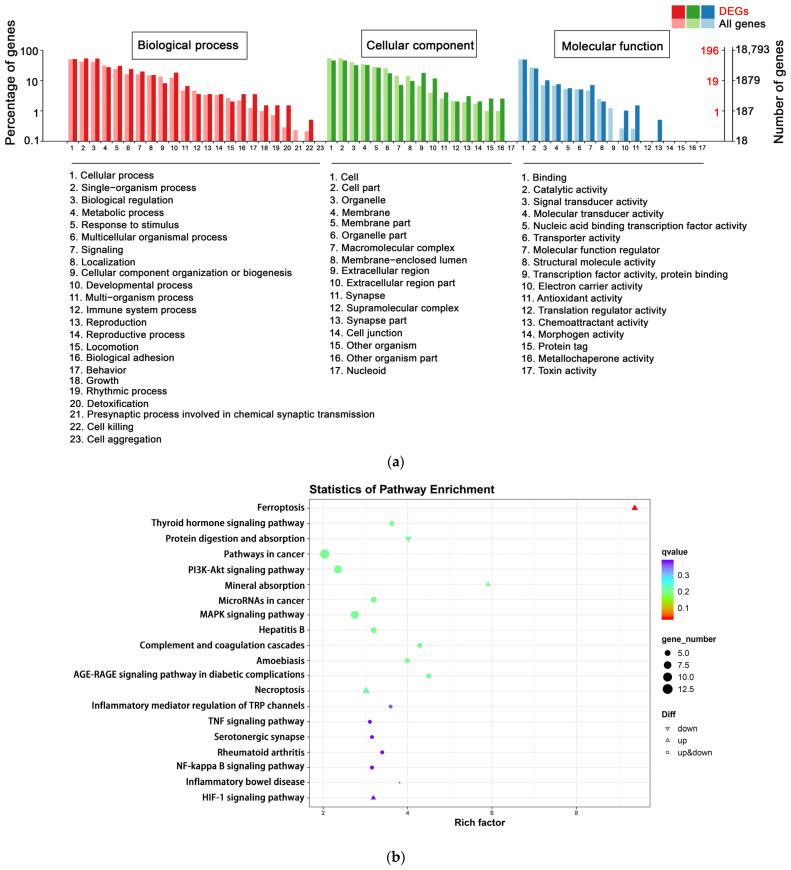
Functional analysis by GO and KEGG enrichment. (**a**) GO classification of DEGs in C vs. CS. The abscissa is the GO classification, the ordinate is the percentage of genes on the left, and the right is the number of genes. Light colors indicate expression across the entire genome, and dark colors indicate expression in DEGs. (**b**) Enrichment of KEGG pathway in DEGs bubble patterns of C vs. CS. Each dot stands for one KEGG pathway. X-axis: enrichment factors; Y-axis: pathway. Enrichment factor = (annotated DEG ratio to overall DEGs)/(annotated gene ratio to overall genes). The dot color represents the q-value (the adjusted *p*-value). When the q value is smaller, enrichment is more reliable and significant. C, cell without treatment; CS, cell treated with 50 µg mL^−1^ SEL.

To better understand the influence of SEL on the biological function of DPCs, GO enrichment analysis was conducted on the following three categories: molecular function, cell composition, and biological process. The terms with two significantly different bars can be picked up as potential targets for further functional analyses, since these GO terms are enriched differently between DEG-based and all-gene-based enrichment. There were 196 DEGs annotated in the C vs. CS group GO enrichment analysis. In biological processes, detoxification (three DEGs), behavior (seven DEGs), and cell killing (one DEG) were significantly different from all-gene-based enrichment. In cellular components, the extracellular region (35 DEGs), other organisms (5 DEGs), and synapse (8 DEGs) were considerably different from all-gene-based enrichment. For molecular function, chemoattractant activity (1 DEG), antioxidant activity (3 DEGs), and electron carrier activity (2 DEGs) were mainly enriched (Figure 3a).

The identified DEGs were analyzed using KEGG to explore the biological functions and interactions of genes. In total, 106 C vs. CS group DEGs were integrated into the KEGG pathway database. The top 20 enriched pathways (with the smallest q-value) are shown in Figure 3b. The top three pathways are ferroptosis (five DEGs), thyroid hormone signaling pathway (five DEGs), and protein digestion and absorption (five DEGs). Notably, DEGs annotated into the ferroptosis pathway were all significantly upregulated. Ferroptosis is a novel regulatory cell death characterized by a high dependence on iron lipid peroxidation and is involved in various biological processes, including the biosynthesis of nicotinamide adenine dinucleotide phosphate (NADPH), oxidative stress, iron and lipid metabolism, glutathione (GSH), and coenzyme Q10 (CoQ10) [48].

### 3.5. Effects of SEL on the Proliferation and Antioxidation of DPC

#### 3.5.1. Changes in the Ferroptosis Signaling Pathway in DPC after SEL Treatment

**Figure 4:**
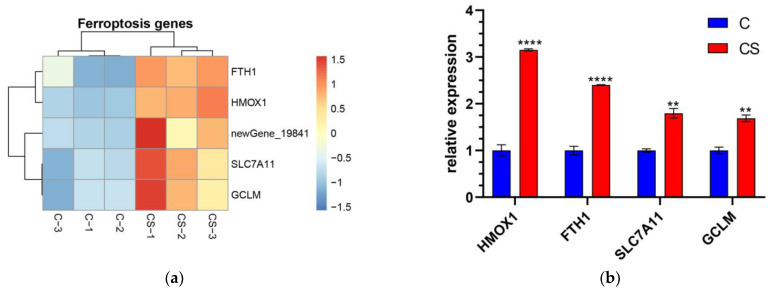

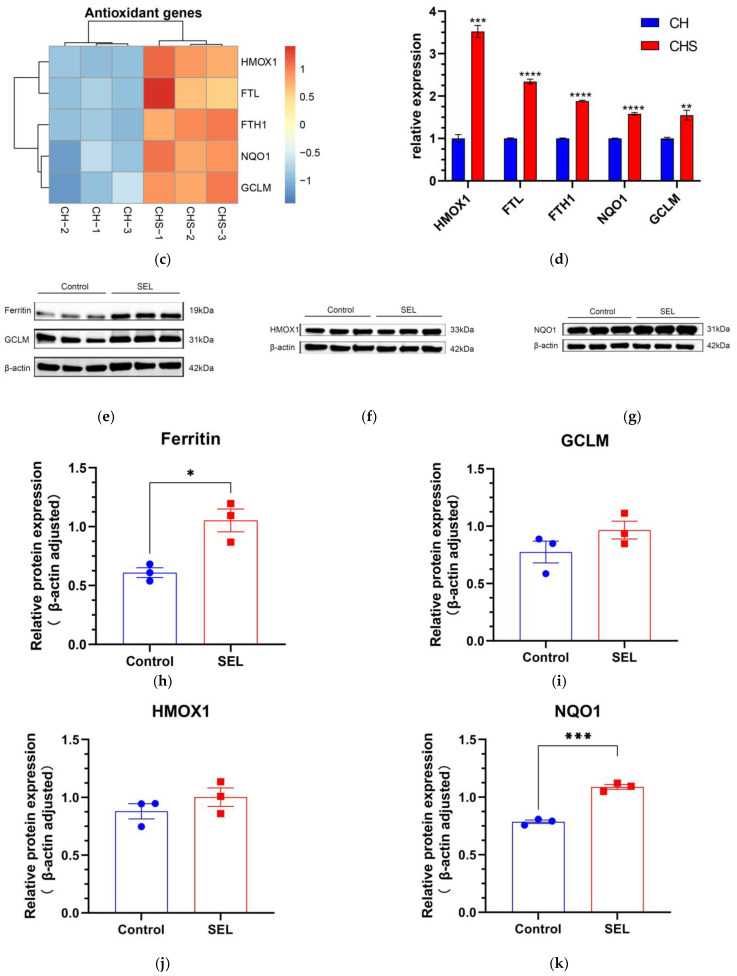
The gene expression level was verified at mRNA and protein levels. (**a**) The heatmap of transcription gene expression levels linked to ferroptosis in C vs. CS; (**b**) RT-qPCR validation of gene expression levels linked to ferroptosis in C vs. CS; C, cell without treatment; CS, cell treated with 50 µg mL^−1^ SEL. (**c**) The heatmap of gene expression levels related to antioxidation in CH vs. CHS; CH, cell treated with 100 µM H_2_O_2_; CHS, cell treated with 50 µg mL^−1^ SEL + 100 µM H_2_O_2_. (**d**) RT-qPCR validation of the transcript expression levels of genes related to antioxidation in CH vs. CHS. (**e**–**k**) The expression level of GCLM, ferritin, HMOX1, and NQO1 was assessed via the Western blotting technique. β-action is used as an internal parameter. The blue dot and the red box represent the protein expression level of the gene in the control group and SEL group, respectively. Each group has three biological replicates. Control, without treatment; SEL, cell treated with 50 µg mL^−1^ SEL for 36 h; HMOX1, heme oxygenase 1; SLC7A11, solute carrier family 7 member 11; FTL, ferritin L subunit; FTH1, ferritin heavy chain 1; NQO1, NAD(P)H quinone dehydrogenase 1; GCLM, glutamate–cysteine ligase modifier subunit. All data presented as the mean ± standard error of the mean (SEM) (*n* = 3). * *p* < 0.05, ** *p* < 0.01, *** *p* < 0.001, and **** *p* < 0.0001 vs. control.

KEGG pathway analysis shows that the ferroptosis signaling pathway was significantly influenced by SEL. A total of five genes in ferroptosis were upregulated after the SEL treatment, including *GCLM, HMOX1, SLC7A11, FTH1*, and Homo_sapiens_newGene_19841 (Figure 4a). The glutamate–cysteine ligase modifier subunit (GCLM, ENSG00000023909) is one of the key subunits of γ-glutamyl cysteine synthase and is the first-rate limiting enzyme for GSH synthesis. Ferroptosis is controlled by the composition of glutathione peroxidase 4 (GPX4). GSH acted as the reaction substrate of GPx4, and its depletion, could cause ferroptosis [49]. Solute Carrier Family 7 Member 11 (SLC7A11, ENSG00000151012) is an amino acid transporter that exchanges _L_-cysteine and _L_-glutamic acid. It plays an integral role in maintaining intracellular GSH levels and cysteine/cysteine extracellular redox balance [50]. Ferritin with light and heavy chains is a widely expressed pathway containing highly conserved proteins. Both light and heavy chains are crucial for maintaining iron homeostasis. Ferritin heavy chain 1 (FTH1, ENSG00000167996) is a key subunit of ferritin that plays a major role in maintaining the cellular iron balance in ferroptosis [51]. It is known that heme oxygenase 1 (HMOX1, ENSG000000100292) can decompose heme and is believed to protect cells under various stress conditions [52]. In addition, a novel gene Homo_sapiens_newGene_19841 in ferroptosis was found, but its specific role remains to be explored. The RT-qPCR results further verified the transcriptional differences of these critical genes. After the SEL treatment, the expression levels of GCLM, HMOX1, SLC7A11, and FTH1 were 1.69, 3.15, 1.80, and 2.40 times that of the control group, respectively (Figure 4b). These results indicated that SEL could significantly affect the ferroptosis process of DPC.

#### 3.5.2. Effect of SEL on the Antioxidant Capacity of DPC

Previous studies reported that oxidative stress causes DPC senescence [53]. RNA-Seq showed that SEL significantly promoted the expression of some antioxidant genes (*HMOX1*, *FTH1*, *FTL*, *NQO1*, and *GCLM*) under oxidative stress (Figure 4c). The expression of genes among two groups was explored by RT-qPCR. Heme acts as a pro-oxidant catalyst to promote further ROS production, while HMOX1 can decompose heme and reduce oxidative damage caused by heme [54]. Ferritin, consisting of ferritin light chain (FTL, ID: ENSG00000087086) and ferritin heavy chain 1 (FTH1, ID: ENSG00000167996), is an indirectly acting antioxidant that inhibits H_2_O_2_ from forming HO^-^ via Fenton chemical reactions. [55]. The most abundant antioxidant within all cells is GSH [56]. GSH is a tripeptide molecule that can protect cells from cell damage caused by oxidative stress and detoxify exogenous substances [57]. GCLM is one of the key subunits of the first rate-limiting enzyme of GSH. NADPH quinone oxidoreductase 1 (NQO1, ENSG00000181019) can catalyze the dual electron reduction in quinone compounds, thereby preventing ROS production and protecting cells from oxidative damage [58]. RT-qPCR results showed that, compared with the control group, SEL significantly increased the expression levels of HMOX1, FTL, FTH1, NQO1, and GCLM of DPC under oxidative stress, which were 3.52, 2.34, 1.88, 1.58, and 1.55 times the control group, respectively (Figure 4d). These results indicated that SEL could improve DPC’s antioxidant capacity and reduce hair follicle damage caused by oxidative stress.

The genes GCLM, ferritin, HMOX1, and NQO1 are essential in ferroptosis and antioxidation. Therefore, the effect of these genes after the DPC treatment with SELs was elucidated via Western blotting. The results show that, compared to the control group, SEL significantly induced the expression of GCLM, ferritin, HMOX1, and NQO1 by 24.8%, 72.7%, 13.9%, and 38.5%, respectively (Figure 4e–k). This was consistent with RT-qPCR results, which further proved that SEL inhibited ferroptosis and improved antioxidant capacity by affecting the expression of these genes.

### 3.6. SEL Promoted Hair Growth in C57BL/6 Mice

**Figure 5:**
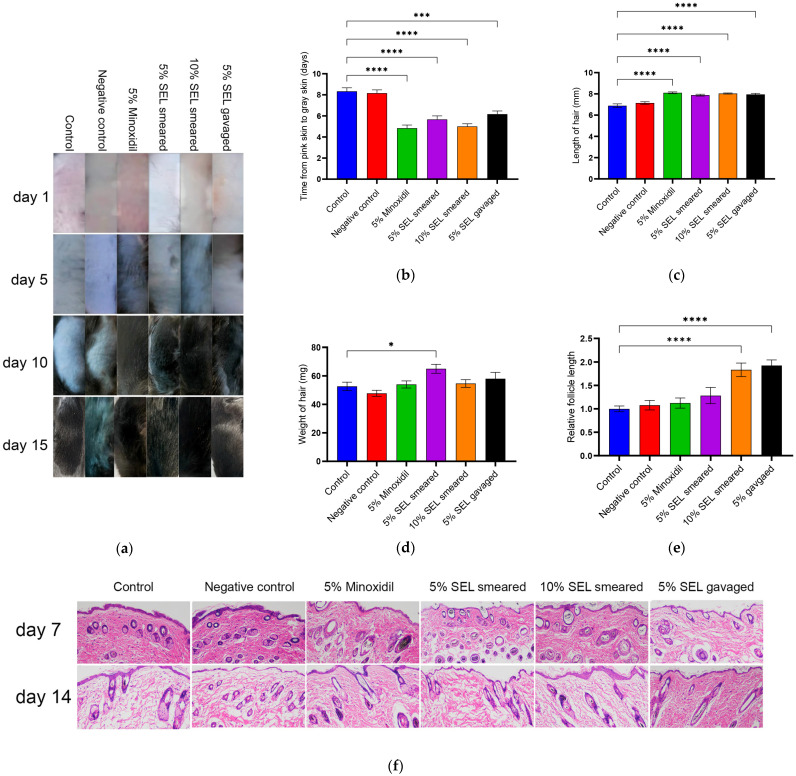
SEL promotes mouse hair growth and hair follicle growth. (**a**) The changes in mice hair after SEL administration. Vehicle (propylene glycol/ethanol (1:1, *v/v*)), 5% SEL, 10% SEL, feed containing 5% SEL, and 5% minoxidil were administered daily for two weeks. (*n* = 5). (**b**) Length of telogen in each group. When the skin of C57BL/6 mice is pink, the hair follicle is considered to be in the telogen period, and when the skin turns gray, it is thought to enter the growth period. (**c**) Length of new hair. (**d**) Weight of new hair. (**e**) The longest hair follicle length on mouse skin (magnification, 100×) (*n* = 3). (**f**) The HE-stained micrographs of skin tissue obtained on days seven and fourteen from mice treated with vehicle, SEL, or minoxidil (*n* = 3). Magnification: 100×. All data presented as the mean ± standard error of the mean (SEM) (*n* = 3). * *p* < 0.05, *** *p* < 0.001, and **** *p* < 0.0001 vs. control.

Currently, the commonly used drug for hair loss on the market is 5% minoxidil [13,59]; thus, we chose 5% SEL, including those smeared and gavaged for efficacy comparison. In addition, we also set a group of smeared 10% SEL to explore the effect of relatively high concentrations of SEL on the hair growth of C57BL/6 mice. Thus, in this study, 5% minoxidil was set as the positive control, and solvent (propylene glycol/absolute ethanol (1:1, V/V)) was set as the negative control. Compared to the control group, the gavage and smear administration of SEL significantly promoted hair regeneration in mice (Figure 5a). The time when the skin of mice changes from pink to gray comprises the telogen of hair follicles [60]. Compared with the control group, the telogen of the negative control group has no significant difference, indicating that the solvent does not affect the hair growth of mice. It is worth noting that SEL significantly shortened the telogen of hair follicles by 2.16–3.33 days compared with the control group (Figure 5b). Moreover, the new hair length of SEL-treated C57BL/6 mice was 14.4%–16.7% longer than the control group (Figure 5c). Regarding the weight of new hair, the 5% SEL smeared group dramatically increased by 23.4% compared with the control group (Figure 5d). New hair follicle length statistics showed that the new hair follicle length after the SEL treatment was 83.4%–92.6% higher than the control group and 70.1%–78.6% higher than the minoxidil group (Figure 5e,f). These results validate that SEL has a hair-growth-promoting property that is similar to minoxidil.

## 4. Discussion

Alopecia has become a major problem that plagues modern people. New drugs are needed to treat hair loss due to the substantial side effects and poor effectiveness of available medications. Although PUFA has been proven to be an essential nutrient the body requires and plays an important role in improving memory, lowering blood pressure, and promoting blood microcirculation, their role in promoting hair growth has rarely been studied. DPC is the core that connects and controls the entire hair follicle cell population; thus, regulating DPC proliferation is crucial for hair growth [61]. In this study, we observed that SEL not only promotes the proliferation of DPCs, but also significantly enhances their antioxidant capacity. In addition, we demonstrated that SEL exerts its effects by promoting the expression of antioxidant genes and inhibiting ferroptosis.

**Figure 6:**
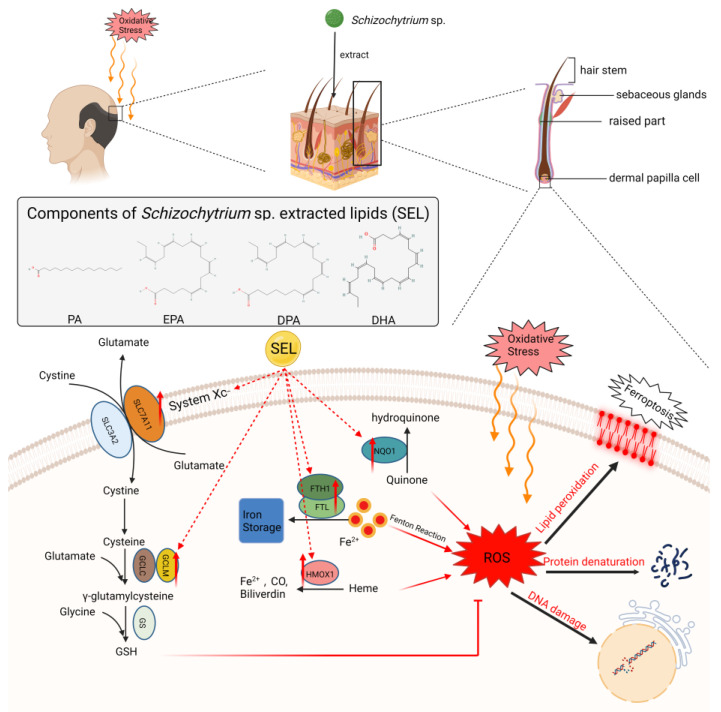
SEL inhibited the occurrence of ferroptosis in DPC and increased its antioxidant capacity. SEL reduces the ROS level in DPC by promoting the expression of SLC7A11, GCLM, HMOX1, NQO1, FTH1, and FTL, which can reduce the damage of lipid peroxidation, protein denaturation, and DNA damage caused by excess ROS. SEL: *Schizochytrium* sp. extracted lipids; SLC7A11: solute carrier family 7 member 11; SLC3A2: solute carrier family 3 member 2; GCLM: glutamate–cysteine ligase modifier subunit; GCLC: cysteine ligase catalytic subunit; GSH: glutathione; GS: glutathione synthetase; FTH1: ferritin heavy chain 1; FTL: ferritin light chain; HMOX1: heme oxygenase 1; NQO1: NAD(P)H quinone dehydrogenase 1.

Oxidative stress is commonly related to local and systemic aging and extensive local health, which may be a key inducer of hair loss [53,62]. Previous research found that oxidative stress increased in young patients with early-onset androgenetic alopecia (AGA) [63]. In addition, studies have shown that the activities of superoxide dismutase (SOD) and glutathione peroxidase (GSH-Px) in red blood cells and total antioxidant capacity in serum are reduced in patients with alopecia areata, supporting a possible role of oxidative stress in the pathogenesis of alopecia areata [7,8]. Interestingly, this study found that SEL (100 µg mL^−1^) can significantly improve the survival rate of DPC against oxidative stress, which is 2.45 and 2.11 times that of the control group and minoxidil group. In addition, SEL can greatly reduce the intracellular ROS level caused by H_2_O_2_. This indicated that SEL could significantly improve the antioxidant activity of DPC. RT-qPCR and WB results exhibited that SEL increased the mRNA and protein levels of antioxidant genes (HMOX1, FTH1, FTL, GCLM, and NQO1) compared to the control group, which may have a positive effect on the treatment of hair loss. HMOX1 is an antioxidant enzyme that protects cells from oxidative damage by producing biliverdin and bilirubin antioxidants [64], consuming heme oxidants [65,66], and regulating vascular tension via carbon monoxide production [67]. Heme can be decomposed by HMOX1 into carbon monoxide (CO), biliverdin, and free iron, which can reduce the production of ROS (Figure 6). Moreover, the expression of HMOX1 on the scalp of patients with alopecia areata decreased [68], and it can be assumed that, by increasing HMOX1 expression, SEL can protect DPCs against oxidative damage to prevent the loss of hair and improve nutrient absorption via the vascular system to enhance hair growth. NQO1 is a potent antioxidant enzyme that reduces ROS production by utilizing NADH or NADPH as a hydride donor and by reducing a wide range of quinones into their corresponding hydroquinone using mandatory double-electron reduction [69] (Figure 6). Research showed that the NQO1 protein protects against neuronal cell death induced by oxidative stress [70]. GCLM, FTL, and FTH1 are also essential members of the antioxidant defense system [71,72]. It is known that the oxidative damage inflicted by ROS can cause major injury to human cells (including DPCs), resulting in serious sickness. Our observations provide a novel and exciting direction for treating alopecia. By regulating antioxidant gene (HMOX1, FTH1, FTL, GCLM, and NQO1) expression, the ROS in the DPCs could greatly decreased, thus healing the hair follicle.

Ferroptosis is a unique oxidative-stress-induced cell death pathway characterized by GSH depletion and lipid peroxidation [73], which can induce cell death in several types of tumors [74]. Ferroptosis may also play an important role in hair loss, since lipid peroxidation can also greatly affect the normal growth of hair [75,76]. The research study observed that hydroperolenic acid’ss (lipid peroxides) topical application resulted in early degeneration in the mouse hair cycle and induced the apoptosis of hair follicle cells [77]. Although previous studies showed that PUFAs can induce ferroptosis in tumor cells [78,79,80], our results showed an interesting phenomenon—that PUFA-rich SEL can inhibit ferroptosis in normal human cells, such as DPCs. It was reported that cystine/glutamate antiporter SLC7A11 (xCT) can introduce cysteines for GSH biosynthesis and antioxidant defense [81]. GCLM is a key subunit of the first rate-limiting enzyme in GSH synthesis [82] (Figure 6). In this study, SEL can significantly increase the expression of SLC7A11 and GCLM, thereby promoting the synthesis of GSH and inhibiting ferroptosis, leading to a positive effect on the treatment of alopecia. Ferritin heavy and light chains (FTH1 and FTL) can isolate excess iron in cells in a non-toxic and easily available form [83]. Research studies found that the knockdown of FTH1 in mice intestines promotes ferroptosis and iron overabsorption [84] (Figure 6). Our results show that SEL can promote the expression of FTH and FTL1, helping the maintenance of iron homeostasis and avoiding ferroptosis, which is of great significance for the normal function of DPCs.

However, our work has certain limitations. Firstly, SEL is a mixture with various unsaturated fatty acids and saturated fatty acids. To clarify the key component and the optimal composition compatibility, the SEL can be further separated by chromatography technology with the influence of the single component on preventing alopecia deeply investigated. Secondly, the hair follicle, an independent micro-organ, is composed of cells from multiple sources, such as DPCs, outer root sheath cells (ORSCs), and hair follicle stem cells (HFSCs) [85,86]. Our results only indicated a positive effect of SEL on DPCs, but its effects on the growth and antioxidative capability of ORSCs and HFSCs still remain unknown. Thirdly, except oxidative damage, there are many other factors leading to the alopecia, such as androgen [87]. Androgen has been considered as the vital factor triggering the hair loss of men, thus it is necessary to analyze the impact of SEL on the androgenetic alopecia in the future [88]. We believe that, with the limitation replenished by the future research, the mechanism of how SEL greatly repairs the damaged hair follicles can be systematic elucidated, thus providing a promising novel material for treating alopecia.

## 5. Conclusions

In conclusion, our findings provided strong evidence that SEL administration in alopecia promotes hair growth and prevents hair loss. In vivo experiments with C57BL/6 mice showed that the hair-growth-promoting effect is comparable to that of 5% minoxidil. SEL could restrain ferroptosis and enable DPCs to function normally by adjusting iron homeostasis and encouraging GSH synthesis in DPCs. Moreover, SEL can significantly strengthen the expression of antioxidant genes (*HMOX1, GCLM, FTL, FTH1,* and *NQO1*), reducing the damage caused by oxidative stress to hair follicles. We elucidated the potential treatment of alopecia by using SELs containing a high percentage of PUFAs.

## Data Availability

Transcriptomic data have been deposited into the NCBI database under BioProject ID PRJNA859644 (https://www.ncbi.nlm.nih.gov/bioproject/PRJNA859644, accessed on 18 July 2022).

## Supplementary Materials

The following supporting information can be downloaded at: https://www.mdpi.com/article/10.3390/antiox12071332/s1, Table S1: Summary of the sequencing read alignment to the reference genome; Table S2: The gene specific primers of RT-qPCR; Table S3: Antibody information.
