# Supplementary material for: Schizochytrium sp. Extracted Lipids Prevent Alopecia by Enhancing Antioxidation and Inhibiting Ferroptosis of Dermal Papilla Cells
Source: Antioxidants (Basel). 2023 Jun 23;12(7):1332. doi: 10.3390/antiox12071332 (PMC10375984; doi:10.3390/antiox12071332)
Supplement: Supplementary file 1 [file antioxidants-12-01332-s001.zip › antioxidants-2301060-supplementary.pdf]

Table S1. Summary of the sequencing read alignment to the reference genome.

**Type**	**Total Reads**	**Mapped Reads**	**%≥Q30**	GC Content
C-1	38,330,038	37,039,373 (96.63%)	94.57%	48.54%
C-2	44,091,654	41,740,979 (94.67%)	94.18%	48.04%
C-3	45,131,850	43,446,064 (96.26%)	94.91%	50.27%
CS-1	39,229,008	37,790,938 (96.33%)	94.58%	47.36%
CS-2	52,199,810	50,493,580 (96.73%)	94.86%	48.23%
CS-3	46,658,058	44,934,545 (96.31%)	94.95%	49.39%
CHS-1	45,110,886	42,902,776 (95.11%)	94.03%	48.35%
CHS-2	42,234,266	40,746,954 (96.48%)	94.79%	48.16%
CHS-3	44,496,668	42,926,550 (96.47%)	94.91%	47.50%
CH-1	42,496,126	41,123,328 (96.77%)	94.98%	47.61%
CH-2	42,939,662	41,528,889 (96.71%)	94.79%	47.24%
CH-3	51,976,310	50,321,083 (96.82%)	94.56%	46.34%

Table S2. The gene specific primers of RT-qPCR.

Gene name	Sequences(5’-3’)	ID	Product Length (bp)
HMOX1	F:TCCAGCTCTTTGAGGAGTTGC<br>R:CTGAGTGTAAGGACCCATCGG	ENSG00000100292	193
FTL	F:CTACACCTACCTCTCTCTGGGCTTC<br>R:TCATGGCGTCTGGGGTTTTAC	ENSG00000087086	209
FTH1	F:ACGTTTACCTGTCCATGTCTTAC<br>R:CTGCAGCTTCATCAGTTTCTC	ENSG00000167996	125
NQO1	F:CGACCTTGTGATATTCCAGTTCC<br>R:CTTTGTCATACATGGCAGCGT	ENSG00000181019	122
GCLM	F:GTCAGGGAGTTTCCAGATGTC<br>R:TCAATAGGAGGTGAAGCAATG	ENSG00000023909	212
SLC7A11	F:CAGGTTATTCTATGTTGCGTCTC<br>R:ATTATCATTGTCAAAGGGTGC	ENSG00000151012	120
ACTB	F:TTAGTTGCGTTACACCCTTTCTTG<br>R:TGCTGTCACCTTCACCGTTCC	ENSG00000075624	157

Table S3. Antibody information

Supplier	Catalog No.	Product Description
Abcam	ab68477	Anti-Heme Oxygenase 1antibody [EPR1390Y]
Abcam	ab126704	Anti-GCLMantibody [EPR6667]
Abcam	ab80588	Anti-NQO1antibody [EPR3309]
Abcam	ab75973	Anti-Ferritin antibody [EPR3004Y]
CST	4970T	β-Actin (13E5) Rabbit mAb
